# 

**Published:** 1887-03-12

**Authors:** 


### THE HOSPITAL

An Institution, Family, and Parochial Journal of Hospitals, Asylums, & all Agencies for the Care of the Sick, Criticism & News.

### CONTENTS.

**Mischievous Misstatements**: a Word of Counsel to the City Companies - 395
**Words of Consolation.**—XXIV. Nurses' Trials—Responsibility - 400
**The Story of the Hospitals.**—The Central London Throat and Ear Hospital - 397
**Nurse Farms, Hospitals, and Private Nursing Institutions.**—The Views of Patients, Superintendents, and Private Nurses - 399
**In the Children's Ward.**—Chapter II. By Walter J. Eccott - 401
**Notes and News.**—Meeting of the Hospitals Association.—Lectures at the Midwives' Institute.—The West London Hospital.—The Hampstead Home Hospital.—Hospital Collecting-box Thefts. — The Queen and the East-end Hospitals. — The City of London Lying-in Hospital.—Vacancies.—The Ashburton and Buckfastleigh Cottage Hospital.—The Burnley Hospital.—Donations and Legacies to Medical Charities. — The Birmingham General Hospital. — Hospitals and the Jubilee Celebration, etc., etc. - 402
**Annotations.**—Newcastle leads the Van.—Annuities for a Term of Years.—Chemists and Druggists. - 405
**A National Pension Fund for Hospital Officials and Nurses.** By Secretaries, Sisters, Nurses, and Others - 406
**A World of Babies.** By One of the Crowd - 407
**Jonah and the Whale**: a True Shark Story - 409
**Disorders of Digestion.**—The Stomach - 409
**In- and Out-Patients' Hospital Diary** - 410
**The Children's Corner.**—Puzzles, Answers, etc. - vi
**Amusements with Profit** - vi

**Hospitals urgently needing Annual Subscriptions, Donations, and Legacies.**

